# The effect of head coil configuration and channel count on the quality of double inversion recovery (DIR) MRI images

**DOI:** 10.1186/s12880-026-02377-1

**Published:** 2026-05-05

**Authors:** Adnan Alahmadi, Razan A. Alshehri, Rana A. Gasem, Abdullah Aljuhani, Almotazbillah Bedaiwi, Afnan A. Malaih, Jamaan Alghamdi, Amal Alsalamah, Shyma M. Alkhateeb, Ghouth Waggass, Mohammad Khalil, Mustafa S. Alhasan, Khalid M. Alshamrani, Ali M. Hendi, Njoud Aldusary, Walaa Alsharif, Norah Y. Hakami, Ibrahem Hussain Kanbayti

**Affiliations:** 1 Department of Radiologic Sciences, Faculty of Applied Medical Sciences, King Abdulaziz University, Jeddah, Saudi Arabia; 2 NMR Research Unit, Queen Square Multiple Sclerosis Centre, Department of Neuroinflammation, UCL Queen Square Institute of Neurology, Faculty of Brain Sciences, University College London, London, UK; 3 Radiological Sciences, Batterjee Medical College, Jeddah, Saudi Arabia; 4 Dr. Sulaiman Alhabib Medical Group, Jeddah, Saudi Arabia; 5 Department of Radiology, King Faisal Specialist Hospital and Research Center, Jeddah, Saudi Arabia; 6 Department of Radiology, Faculty of Medicine, King Abdulaziz University, Jeddah, Saudi Arabia; 7 Internal Medicine, Taibah University, Madinah, Saudi Arabia; 8 College of Applied Medical Sciences, King Saud Bin Abdulaziz University for Health Sciences, Jeddah, Saudi Arabia; 9 King Abdullah International Medical Research Centre, Jeddah, Saudi Arabia; 10 Ministry of the National Guard - Health Affairs, Jeddah, Saudi Arabia; 11 Department of Radiology College of Medicine, Jazan University, Jazan, Saudi Arabia; 12 Department of Diagnostic Radiology, College of Applied Medical Sciences, Taibah University, Madinah, Saudi Arabia

**Keywords:** Magnetic resonance imaging (MRI), Double inversion recovery, (DIR), Image quality, Signal-to-noise ratio, 64-channel head coil, 20-channel head coil

## Abstract

**Background:**

Double inversion recovery (DIR) MRI provides high sensitivity for detecting white matter abnormalities but suffers from reduced signal-to-noise ratio (SNR) due to simultaneous suppression of multiple tissue signals. Head-coil configuration and channel count may influence the resulting image quality.

**Methods:**

Seventeen healthy subjects underwent DIR imaging on a 3-T MRI system using both 64-channel and 20-channel head/neck coils. Quantitative image quality was assessed using SNR and contrast-to-noise ratio (CNR) measurements across multiple brain regions, with comparisons performed using paired t-tests. Structural Similarity Index Measure (SSIM) was additionally computed between registered 64-channel and 20-channel DIR images to quantify inter-coil structural image similarity. Qualitative image quality was evaluated by three experienced neuroradiologists using a 5-point rating scale for contrast, spatial resolution, and noise; inter-rater agreement was assessed using Kendall’s coefficient of concordance (Kendall’s W).

**Results:**

Quantitative analysis demonstrated significantly higher SNR and CNR values for the 64-channel coil compared with the 20-channel coil across all assessed regions (*p* < 0.0001). Qualitative evaluation showed that images acquired with the 64-channel coil received marginally higher mean scores for contrast, spatial resolution, and noise from all raters; inter-rater agreement was moderate-to-strong across all domains (Kendall’s W = 0.33–0.89).

**Conclusion:**

At 3 T, the use of a 64-channel head/neck coil provides significant quantitative improvements in DIR image quality compared with a 20-channel coil, with small but consistent advantages also observed in qualitative assessments. These findings support the use of higher-channel-count coils to mitigate SNR limitations inherent to DIR imaging. However, qualitative differences between coil configurations were modest and inter-rater agreement was moderate-to-strong by Kendall’s W (W = 0.33–0.89). The clinical benefit of the 64-channel coil in pathological conditions such as multiple sclerosis or cortical dysplasia requires further investigation in patient-based studies.

## Introduction

Magnetic resonance imaging (MRI) is a diagnostic imaging modality that utilizes a strong magnetic field to generate images with superior soft-tissue contrast and precise tissue characterization [1, 2]. Over the years, MRI has undergone substantial development driven by continuous advancements in hardware and software technologies. As a result, it has become the gold standard for many clinical applications in neurological imaging [3].

One of the essential hardware components in MRI systems is the radiofrequency (RF) coil, which is responsible for transmitting RF pulses to excite proton spins and receiving the emitted MR signals [4]. RF coils directly influence signal-to-noise ratio (SNR), contrast-to-noise ratio (CNR), and spatial resolution, making them a critical determinant of overall image quality [5]. Recent advancements in RF coil design have enabled the development of phased-array head coils with an increased number of receiver channels, leading to improvements in several image quality parameters [6]. Numerous studies have demonstrated that increasing the number of coil channels can significantly enhance image quality [7, 8].

Different tissue contrasts in MRI can be achieved by adjusting radiofrequency pulse timing and sequence parameters. The introduction of inversion recovery (IR) sequences enabled selective suppression of specific tissue signals through appropriate selection of the inversion time (TI) [9]. Building on this principle, double inversion recovery (DIR) suppresses signals from two tissue types simultaneously, most commonly cerebrospinal fluid (CSF) and white matter (WM) [10, 11]. DIR has demonstrated greater sensitivity than conventional MRI techniques for detecting white matter abnormalities [10]. It has proven useful in identifying demyelinating lesions in multiple sclerosis, malignancies, cortical abnormalities, and epileptogenic foci associated with congenital or acquired neocortical disorders [12]. Accordingly, DIR holds considerable diagnostic value in clinical neuroimaging [13].

However, achieving high isotropic spatial resolution for accurate visualization of the neocortex and small cortical lesions remains challenging due to the inherent reduction in SNR caused by the suppression of multiple tissue signals [14, 15]. Therefore, this study investigates whether increasing the number of head-coil channels can effectively mitigate this SNR reduction and enhance overall DIR image quality, thereby providing clinicians with clearer and more detailed diagnostic information.

## Materials and methods

### Participants

The sample was selected from individuals who met the study inclusion criteria. Adult participants aged 20 to 40 years were included to minimize potential confounding effects related to age-associated brain changes. Subjects with contraindications to MRI were excluded. Participant eligibility was confirmed using a standardized MRI safety questionnaire, and written informed consent was obtained from all subjects prior to imaging. The study protocol was approved by the Research Ethics Committee of the Faculty of Applied Medical Sciences at King Abdulaziz University, Saudi Arabia.

### Data acquisition

The study was conducted in the MRI Laboratory of the Department of Radiologic Sciences, Faculty of Applied Medical Sciences, King Abdulaziz University, Saudi Arabia. All imaging data were acquired using a 3-T MRI scanner (Magnetom Vida, Siemens).

Two commercially available head coils were evaluated: a 20-channel head/neck coil (Head/Neck 20) and a 64-channel head/neck coil (BioMatrix Head/Neck 64). The 20-channel coil consists of 20 loop elements arranged in three rings: two rings containing eight elements each and one ring containing four elements. Its inner vertical and horizontal diameters are 26.5 cm and 23 cm, respectively. The 64-channel coil features an anthropomorphic geometry with 64 loop elements, including 24 elements in the upper portion and 40 elements in the lower portion, with inner vertical and horizontal diameters of 22 cm and 19.5 cm, respectively.

For each participant, two imaging sequences were acquired: T1-weighted MPRAGE (Magnetization Prepared Rapid Gradient Echo), which served as a reference for registration and normalization, and DIR images acquired using both head coils. The total scanning time for each subject was approximately 30 minutes, and data collection was completed over four consecutive days.

**Fig. 1:**
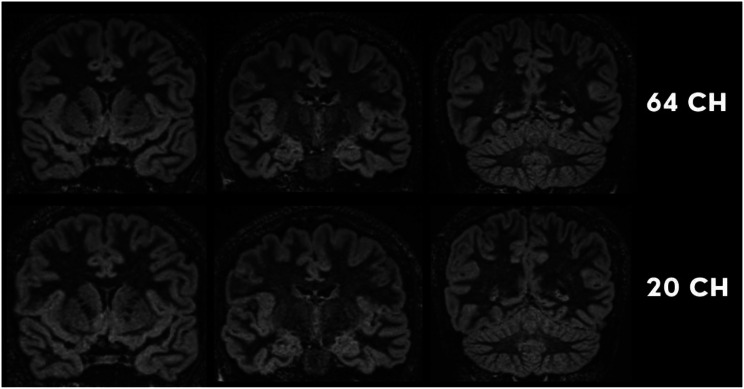
Coronal DIR SPACE sequence of 64-channel Head/Neck coil and 20-channel Head/Neck coil

All participants were scanned first using the 64-channel coil, followed by the 20-channel coil, using identical acquisition protocols and fixed imaging parameters (Table 1). Parallel imaging was applied for both coils with an acceleration factor of 2 to maintain clinically acceptable scan times, consistent with previous studies [16]. The SPACE (Sampling Perfection with Application optimized Contrasts using different flip-angle Evolutions) technique was selected for DIR acquisition due to its rapid imaging capability and demonstrated improvements in SNR, CNR, and spatial resolution [17] (Fig. 1).

**Table 1:** Description of sequence parameters

Parameters	DIR SPACE	T1 mprage p2 iso
Slices	176	176
Phase Oversampling	0%	0%
Slice Oversampling	18.2%	18.2%
FoV Read	255 mm	255 mm
FoV Phase	100%	100%
Slice Thickness	1 mm	1 mm
TR	7500 ms	1750 ms
TE	318 ms	2.26 ms
TI1	3000 ms	900 ms
TI2	450 ms	
Flip Angle		8°
Matrix	256 × 256	256 × 256
Voxel Size	1 mm	1 mm

It should be noted that background noise-based SNR estimation was employed throughout this study, in which SNR is defined as the ratio of regional signal intensity to the standard deviation of background air noise. While widely applied and standardised, this approach may not fully account for the spatially non-uniform noise amplification introduced by parallel imaging. The g-factor associated with an acceleration factor of 2 varies across brain regions and between coil configurations, and may result in regional overestimation of true SNR [18]. This represents a methodological limitation that should be considered when interpreting the quantitative findings.

### Data analyses image processing

Image preprocessing was performed using the Statistical Parametric Mapping (SPM12) software implemented in MATLAB (R2023b). DIR images were first converted from DICOM to NIfTI format. Each DIR image was then realigned and registered to the corresponding T1-weighted MPRAGE image of the same subject. Spatial normalization was subsequently applied to align images acquired with both head coils into identical stereotaxic coordinates to enable accurate quantitative comparison.

Spatial normalization involved deforming gray-matter images into a standardized stereotaxic space by matching them to a common template [19]. Four anatomical regions of interest (ROIs) were initially identified using the SPM Anatomy Toolbox (MATLAB) and MRIcron software. For signal quantification, square ROIs were manually placed using MIPAV software over representative areas of the thalamus, cerebellum, motor cortex, orbitofrontal cortex (OFC), white matter (WM), and background noise regions. Identical ROIs of equal size were positioned at corresponding anatomical locations and slice levels for each subject and coil configuration.

### Quantitative analysis

**Fig. 2:**
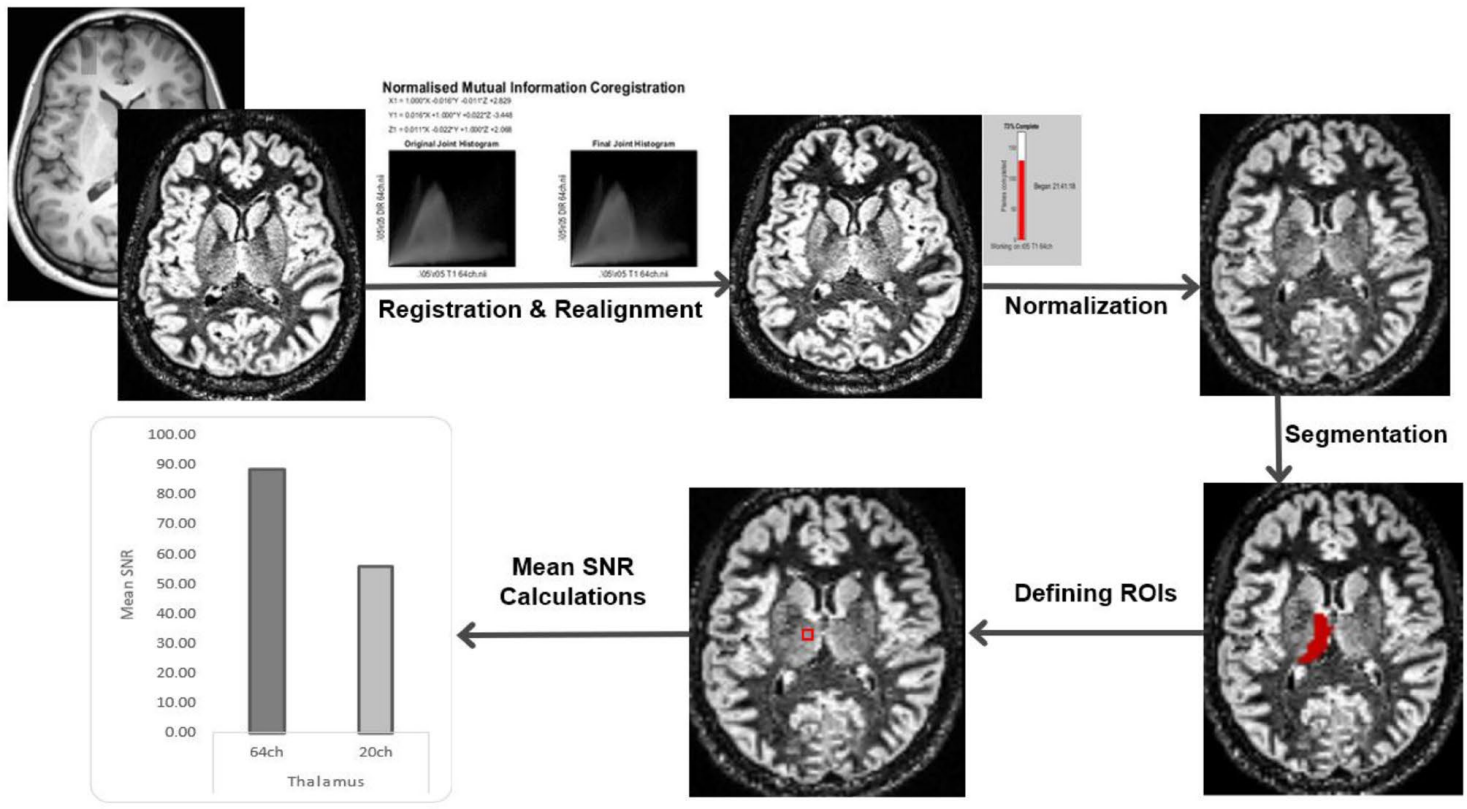
An illustration of methodology. DIR images are aligned and registered to the T1-weighted MPRAGE and subsequently normalised. A square ROI (thalamus example shown) is placed to measure signal intensity and background noise

Group-level quantitative analysis was performed by comparing ROI-based measurements obtained from DIR images acquired using both coils. SNR was calculated as the ratio of signal intensity (SI) to the standard deviation of background noise (SD$_{no}$) (Eq. 1). CNR between gray matter (OFC) and white matter was calculated as the absolute difference between their signal intensities divided by SD$_{no}$ (Eq. 2). The analysis workflow is illustrated in Fig. 2.

Statistical comparisons were conducted using paired two-tailed t-tests to evaluate differences in SNR and CNR between coil configurations, with statistical significance defined as *p* < 0.05.

Effect sizes were quantified using Cohen’s d for paired samples, calculated as d = M_diff / SD_diff, where M_diff is the mean paired difference between coil conditions and SD_diff is the standard deviation of those differences. Ninety-five percent confidence intervals (CIs) for Cohen’s d were derived using the non-central t-distribution method. Post-hoc statistical power was estimated for each comparison using a non-centrality parameter approach (α = 0.05, two-tailed, *n* = 14).

1 $${\rm{SNR}} = {{SI} \over {S{D_{no}}}}$$

2 $${\rm{CNR}} = {{\mid S{I_1} - S{I_2}\mid } \over {S{D_{no}}}}$$

### Qualitative analysis

For qualitative evaluation, five subjects were randomly selected. For each subject, two DIR images acquired using the 20-channel and 64-channel coils were assessed. Three experienced neuroradiologists, each with more than 10 years of clinical experience, independently evaluated the images.

To minimize observer bias, the coil types were anonymized and labeled as “A” (64-channel) and “B” (20-channel). The evaluation survey consisted of three items assessing contrast, spatial resolution, and noise, each rated on a 5-point Likert scale. Scores of 3 or higher were classified as indicative of good image quality, whereas scores below 3 were considered poor. Mean scores were calculated for each rater and subsequently averaged to obtain overall mean scores for each image quality parameter and coil type. Inter-rater agreement among the three raters was assessed using Kendall’s coefficient of concordance (Kendall’s W), a non-parametric measure appropriate for ordinal rating data from multiple raters. The strength of agreement was interpreted according to the magnitude of W: values below 0.30 indicate weak agreement, 0.31–0.50 moderate agreement, 0.51–0.70 good agreement, and values above 0.70 indicate strong agreement. Statistical significance was evaluated using the associated chi-square test, with *p* < 0.05 considered statistically significant. Quantitative statistical analyses (SNR, CNR, Cohen’s d, and post-hoc power) were performed using IBM SPSS Statistics, while Kendall’s W and associated chi-square statistics were computed using Python (version 3.x; SciPy library, version 1.x) [20].

## Results

### Quantitative analysis Results

The study initially included 17 healthy volunteers (nine males and eight females) aged between 20 and 27 years, with a mean age of 22.18 ± 1.63 years. Three participants were excluded due to excessive motion artifacts. Consequently, data from 14 subjects were included in the final statistical analysis.

**Fig. 3:**
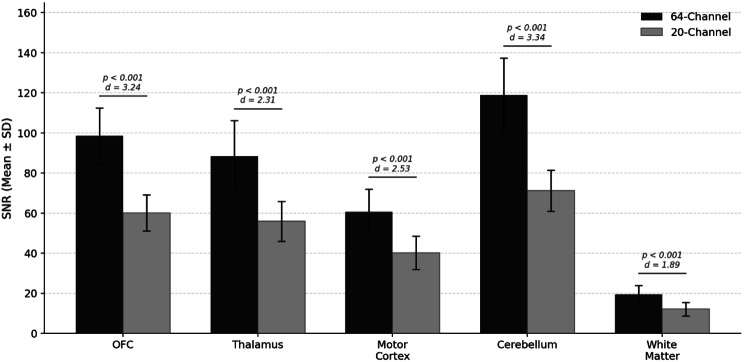
Mean SNR ± SD for 64-channel and 20-channel coils across five brain regions including white matter (WM), with paired t-test *p*-values and Cohen’s d effect sizes annotated. All comparisons: *p* < 0.0001

Comparative analysis of SNR between the 20-channel and 64-channel head/neck coils demonstrated significant differences across all evaluated brain regions (Fig. 3). In every region examined, SNR values were substantially higher when using the 64-channel coil compared with the 20-channel coil. The mean SNR in the orbitofrontal cortex (OFC) was 98.62 ± 13.88 for the 64-channel coil and 60.12 ± 9.08 for the 20-channel coil. In the thalamus, mean SNR values were 88.30 ± 17.86 and 55.89 ± 10.02, respectively. For the motor cortex, mean SNR values were 60.70 ± 11.23 for the 64-channel coil and 40.23 ± 8.31 for the 20-channel coil. In the cerebellum, mean SNR values were 118.87 ± 18.58 and 71.18 ± 10.27 for the 64-channel and 20-channel coils, respectively. All differences in mean SNR values were statistically significant (*p* < 0.0001). White matter SNR was also significantly higher for the 64-channel coil (19.40 ± 4.53) compared with the 20-channel coil (12.04 ± 3.36; d = 1.89, 95% CI: 1.01–2.76, *p* < 0.0001).

Effect size analysis confirmed very large paired Cohen’s d values for all brain regions: orbitofrontal cortex (d = 3.24, 95% CI: 1.93–4.55), thalamus (d = 2.30, 95% CI: 1.30–3.31), motor cortex (d = 2.53, 95% CI: 1.45–3.60), cerebellum (d = 3.34, 95% CI: 2.00–4.69), and white matter (d = 1.89, 95% CI: 1.01–2.76). Post-hoc power analysis confirmed that all comparisons achieved statistical power of 1.00 (α = 0.05, two-tailed, *n* = 14), demonstrating that the study was fully adequately powered to detect the observed effect sizes.

**Fig. 4:**
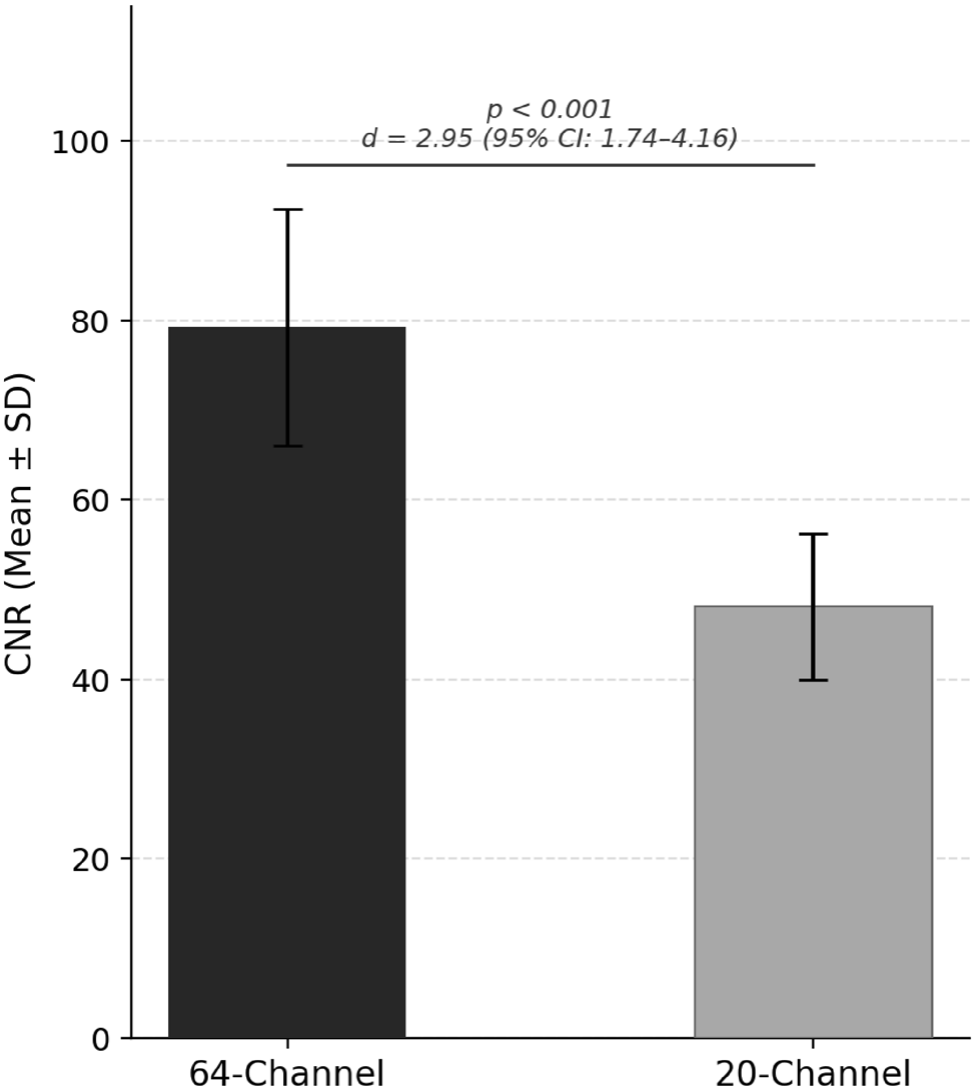
Mean CNR ± SD for 64-channel vs 20-channel coils (*p* < 0.0001, Cohen’s d = 2.95)

Quantitative analysis also demonstrated a significant improvement in gray matter–white matter (GM–WM) CNR when using the 64-channel coil compared with the 20-channel coil. The mean CNR was 79.22 ± 13.19 for the 64-channel coil and 48.08 ± 8.14 for the 20-channel coil. This difference was statistically significant (*p* < 0.0001), indicating superior tissue contrast performance with the 64-channel configuration (Fig. 4).

The CNR comparison yielded a large effect size (d = 2.95, 95% CI: 1.74–4.16), with post-hoc power of 1.00, confirming excellent statistical sensitivity for detecting the observed CNR difference.

### Qualitative analysis Results

**Fig. 5:**
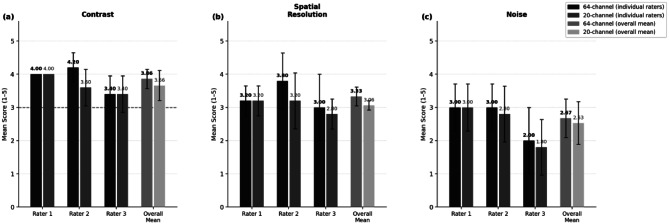
Mean qualitative image quality scores for (**a**) contrast, (**b**) spatial resolution, and (**c**) noise, by rater (rater 1, rater 2, rater 3) and overall mean for 64-channel and 20-channel Head/Neck coils. In panel (**a**), the dashed line marks the good/poor quality threshold (score ≥ 3). All panels share the same legend (upper right)

Qualitative assessment revealed no statistically meaningful differences in ratings between the two coil configurations across evaluators. It should be noted that these qualitative findings are based on only five subjects and should be interpreted with caution given the moderate-to-strong inter-rater agreement indicated by Kendall’s W (W range: 0.33–0.89). For image contrast, the overall mean score ± SD was 3.86 ± 0.29 for the 64-channel head/neck coil and 3.66 ± 0.45 for the 20-channel head/neck coil (Fig. 5a).

For spatial resolution, both coils demonstrated comparable performance, with mean scores of 3.33 ± 0.28 for the 64-channel coil and 3.06 ± 0.15 for the 20-channel coil (Fig. 5b).

Similarly, image noise ratings were comparable between the two configurations. The mean score for the 64-channel coil was 2.67 ± 0.58, while the mean score for the 20-channel coil was 2.53 ± 0.64, indicating a similar perceived level of noise for both coils (Fig. 5c).

**Fig. 6:**
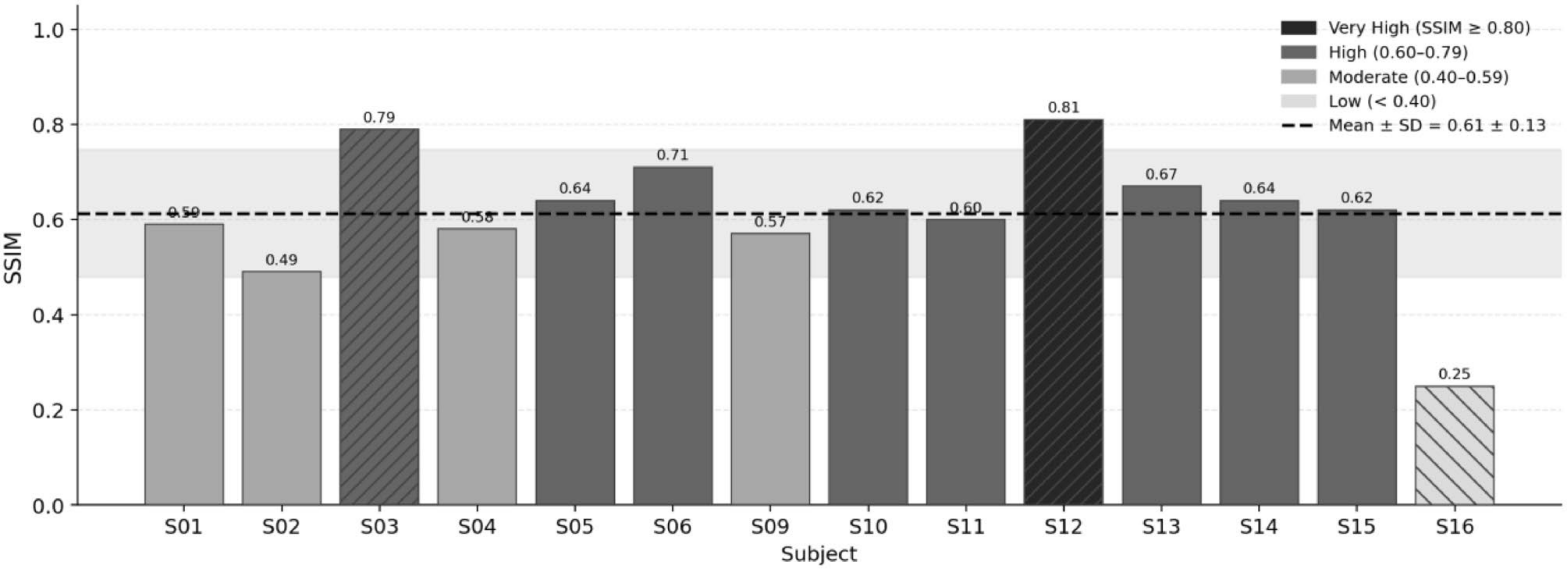
Structural similarity Index Measure (SSIM) between spatially registered 64-channel and 20-channel Double inversion recovery (DIR) images for all 14 included subjects. The dashed line indicates the group mean SSIM (0.61 ± 0.13); the shaded band represents ±1 SD. Values range from 0.25 to 0.81. Subject 16 (SSIM = 0.25) demonstrated notably lower similarity, likely attributable to residual motion artefact or registration imprecision

Inter-rater agreement, assessed using Kendall’s coefficient of concordance (Kendall’s W), indicated moderate-to-strong agreement across all three image quality domains. For the contrast domain, moderate agreement was observed (W = 0.33, χ^2^  = 6.60, *p* = 0.03), indicating a statistically significant consistent ranking pattern among raters. Similarly, spatial resolution demonstrated moderate agreement (W = 0.38, χ^2^  = 7.78, *p* = 0.02), suggesting general consistency in rater evaluations despite some variability in scoring. The noise domain showed strong agreement (W = 0.894, χ^2^  = 17.8, *p* < 0.0001), reflecting a high level of consensus among raters when assessing noise characteristics. These findings indicate that noise may be a more consistently perceived image quality parameter, while contrast and spatial resolution involve greater inter-rater subjectivity (Fig. 6).

**Fig. 7:**
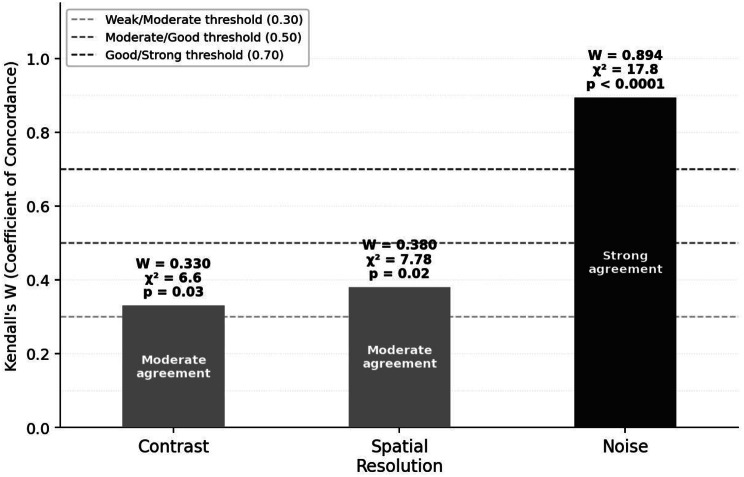
Kendall’s coefficient of concordance (W) for inter-rater agreement across three image quality domains (contrast, spatial resolution, noise) assessed by three raters in five subjects. Reference lines indicate agreement thresholds: 0.30 (weak/moderate), 0.50 (moderate/good), and 0.70 (good/strong). Bar values show W with associated χ^2^ and *p*-values. Moderate agreement was observed for contrast (W = 0.33, *p* = 0.03) and spatial resolution (W = 0.38, *p* = 0.02), and strong agreement for noise (W = 0.894, *p* < 0.0001)

To further quantify image similarity between coil configurations, the Structural Similarity Index Measure (SSIM) was computed between spatially registered 64-channel and 20-channel DIR images for all 14 included subjects. The mean SSIM was 0.61 ± 0.13 (range: 0.25–0.81), indicating moderate-to-high overall structural similarity between the two acquisition conditions following spatial normalisation. Seven subjects (50%) achieved high similarity (SSIM 0.60–0.81), five subjects (36%) achieved moderate similarity (SSIM 0.40–0.60), and one subject showed low similarity (SSIM = 0.25), likely attributable to residual motion artifact or registration imprecision in that participant (Fig. 7).

## Discussion

One of the primary clinical advantages of multichannel head coils is their compatibility with parallel imaging techniques, which allow for accelerated scan times without compromising SNR or spatial resolution [16, 21]. In addition, numerous experimental and theoretical studies have demonstrated the benefits of increasing the number of elements in phased-array coils [22, 23]. The addition of coil elements improves signal sensitivity in regions close to the coil, although this benefit decreases with increasing distance from the coil surface [22, 24].

The present study evaluated the performance of two head-coil configurations by quantitatively assessing SNR and CNR and qualitatively evaluating overall image quality. The quantitative results demonstrated that the 64-channel head/neck coil consistently outperformed the 20-channel coil across all examined brain regions, including the orbitofrontal cortex, thalamus, motor cortex, and cerebellum. These findings are consistent with previous reports indicating that higher channel counts improve signal reception efficiency and enable more advanced imaging capabilities [25, 26]. In addition, a significant increase in GM–WM CNR was observed with the 64-channel coil, further supporting its superior tissue contrast performance [26]. High SNR and CNR are essential for detecting subtle pathological changes and delineating fine anatomical structures, which directly impacts diagnostic accuracy and clinical decision-making [16].

Despite these objective improvements, qualitative evaluation revealed only small differences in mean image-quality scores between the two coils, with no statistically significant differences observed. Nevertheless, all neuroradiologists expressed a consistent preference for images acquired using the 64-channel coil across contrast, spatial resolution, and noise criteria. It should be emphasised, however, that this apparent preference is inferred from higher mean Likert scores and does not represent a formally assessed preference task; furthermore, the moderate inter-rater agreement for contrast and spatial resolution (Kendall’s W = 0.33 and 0.38, respectively) should be noted when interpreting this observation, as some rater variability in scoring was observed. The comparable noise levels observed between the two coils may be explained by mutual inductive coupling between nearby coil elements and signal averaging across channels, which can result in noise levels that scale proportionally with the number of coil elements [23, 24, 27].

These findings suggest that although high-channel-count coils such as the 64-channel configuration provide clear theoretical advantages in SNR and parallel imaging performance [28], these benefits do not always translate into large perceptible differences in image quality for all sequences or applications. Previous studies have reported significant improvements in anatomical delineation and diagnostic confidence with higher channel coils for certain MRI sequences [16]. However, subjective image quality assessment does not necessarily correlate directly with quantitative metrics such as SNR and spatial resolution [25], and may depend on the specific imaging protocol and clinical task. Our qualitative findings further support the notion that radiologists’ perception of image quality is sequence-dependent and context-specific.

The SSIM analysis (mean 0.61 ± 0.13) further supports these findings by demonstrating moderate-to-high structural similarity between the two coil configurations after spatial registration, suggesting that the fundamental image information is preserved across coil types despite quantitative differences in SNR and CNR. The inter-subject variability in SSIM (range: 0.25–0.81) may reflect differences in motion susceptibility, coil fit, or registration accuracy, and warrants further investigation in larger cohorts. Additional structural similarity metrics such as the Dice Similarity Coefficient (DSC) and Hausdorff Distance were not computed in the present study, as these require binary segmentation masks that were not generated in the processing pipeline; their application is recommended for future studies.

Several limitations should be acknowledged. First, this study was conducted exclusively in healthy volunteers, preventing assessment of coil performance for lesion detection or diagnostic accuracy in pathological conditions. Earlier investigations that included abnormal brain findings demonstrated that lesion conspicuity and diagnostic performance are critical factors in evaluating coil superiority [10, 16]. Future studies incorporating patients with multiple sclerosis or other white-matter pathologies would provide a more comprehensive evaluation of the clinical utility of DIR imaging with high-channel coils.

A further methodological limitation concerns the fixed acquisition order employed in this study: all participants were scanned first using the 64-channel coil and subsequently with the 20-channel coil. This approach was adopted for practical reasons, specifically to minimise total examination time and reduce the logistical complexity of coil repositioning within a single session. However, fixed ordering introduces the potential for order effects, including participant fatigue, subtle positional changes following coil exchange, and environmental habituation. Although such effects are unlikely to account for the large magnitude of quantitative differences observed, we acknowledge this as a limitation of the study design. Future studies should consider counterbalanced or randomised coil ordering to control for these potential confounds.

It should be explicitly stated that improvements in SNR and CNR demonstrated in healthy volunteers do not necessarily translate to improved lesion detectability or diagnostic accuracy in pathological conditions such as multiple sclerosis, epilepsy, or cortical dysplasia—precisely those disorders for which DIR imaging is most clinically relevant. Higher SNR is a necessary but not sufficient condition for improved clinical diagnosis; patient-based studies incorporating lesion detection sensitivity and specificity, reader confidence, and clinical decision-making outcomes are required to determine whether the quantitative advantages of the 64-channel coil translate into meaningful clinical benefit [13, 29].

Second, qualitative image assessment was performed using static images rather than full diagnostic DICOM viewers, which may not fully reflect routine clinical interpretation conditions. Third, although the 64-channel coil yielded superior SNR and CNR, it exhibited a higher susceptibility to motion artifacts during data acquisition. This may be attributed to its smaller and tighter physical design, which could reduce subject comfort and increase involuntary movement.

Three of the 17 recruited participants were excluded from the final analysis due to motion-related image degradation, and all three exclusions occurred during 64-channel acquisitions. This asymmetric exclusion pattern may suggest that the tighter physical geometry of the 64-channel coil—with smaller inner vertical (22 cm) and horizontal (19.5 cm) diameters compared with the 20-channel coil (26.5 cm × 23 cm)—may reduce subject comfort and thereby increase the propensity for involuntary motion. However, as motion susceptibility was not systematically monitored or quantified across coil conditions in this study, this observation should be interpreted with caution. Future studies should incorporate prospective motion monitoring (e.g., navigator echoes or optical tracking) and directly compare motion metrics across coil configurations to determine whether coil geometry is an independent predictor of motion artifacts in DIR imaging.

Furthermore, the background noise-based SNR estimation used in this study may not fully capture the spatially non-uniform noise amplification introduced by parallel imaging acquisition with an acceleration factor of 2. The g-factor penalty, which varies with coil geometry and brain region, can result in regionally variable SNR that is not adequately represented by a global background noise standard deviation. This may lead to systematic overestimation of SNR in regions with elevated g-factor penalties, particularly in central brain structures for high-channel-count coils [18]. Future studies should consider more rigorous SNR quantification methods such as pseudo-multiple replica or noise covariance matrix-based approaches, which better account for the spatially varying noise characteristics inherent to parallel imaging.

Finally, the relatively small sample size for qualitative evaluation limits the generalizability of the subjective findings. Increasing the number of evaluated cases would strengthen statistical power and clinical relevance. Inter-subject anatomical variability may also influence quantitative measurements and should be considered in future investigations [30, 31].

Furthermore, the qualitative assessment was restricted to five randomly selected subjects, substantially limiting the statistical power and generalisability of the subjective image quality findings. With only *n* = 5 subjects evaluated qualitatively, the study lacked sufficient power to detect small but clinically meaningful differences in perceived image quality between coil configurations. The moderate-to-strong Kendall’s W values (W = 0.33–0.89) reported above indicate that raters showed consistent ranking patterns, particularly for noise assessment; however, the limited sample size (*n* = 5) substantially reduces the generalisability of these findings and highlights the need for formal rater calibration and standardised evaluation criteria in future studies. Future studies should aim to include at least 20–30 subjects for qualitative evaluation, with dedicated rater training to improve inter-observer reliability and the clinical interpretability of subjective assessments.

## Conclusion

At 3 T, the 64-channel head/neck coil provides statistically significant improvements in quantitative DIR image quality, particularly in SNR and CNR, compared with the standard 20-channel coil. Qualitative evaluation demonstrated only modest differences in perceived image quality; however, the 64-channel coil consistently received higher ratings across all evaluated parameters. The increased susceptibility to motion artifacts observed with the 64-channel coil warrants further investigation and may represent a practical limitation in certain clinical settings. Overall, higher channel-count head coils offer measurable technical advantages for DIR imaging, although their impact on subjective image quality and diagnostic performance appears to be sequence- and application-dependent. Additionally, SSIM analysis demonstrated moderate-to-high structural similarity between coil configurations (mean 0.61 ± 0.13), indicating that the fundamental image information is largely preserved across coil types despite the quantitative differences in SNR and CNR observed. Several important caveats apply: qualitative findings are based on only five subjects with moderate-to-strong inter-rater agreement by Kendall’s W (W = 0.33–0.89); the fixed acquisition order may introduce order effects that could partially confound quantitative comparisons; SNR estimates based on background noise may be subject to systematic overestimation due to g-factor effects under parallel imaging; and all participants were healthy volunteers, meaning the clinical benefit of the 64-channel coil for lesion detection in pathological conditions such as multiple sclerosis or cortical dysplasia remains to be established in patient-based studies.

## Data Availability

The datasets generated and analysed during the current study are not publicly available due to patient privacy and institutional data governance requirements, but are available from the corresponding author on reasonable request, subject to appropriate data sharing agreements.

## Abbreviations

DIR: Double Inversion Recovery
MRI: Magnetic Resonance Imaging
SNR: Signal-to-Noise Ratio
CNR: Contrast-to-Noise Ratio
OFC: Orbitofrontal Cortex
WM: White Matter
GM: Grey Matter
ROI: Region of Interest
SD: Standard Deviation
CI: Confidence Interval
SSIM: Structural Similarity Index Measure
3T: 3 Tesla
MPRAGE: Magnetisation-Prepared Rapid Gradient Echo
SPACE: Sampling Perfection with Application-optimised Contrasts using different flip angle Evolutions
MS: Multiple Sclerosis

## Declarations

### Declarations

NA.

## References

[1] Jaspan ON, Fleysher R, Lipton ML. Compressed sensing MRI: a review of the clinical literature’, 2015. BJR. 2015;88(1056):20150487. 10.1259/bjr.20150487. 26402216 10.1259/bjr.20150487 PMC4984938

[2] Bell D, Jones J. MRI. Radiopaedia.org, Radiopaedia.org. 2009. 10.53347/rID-6317.

[3] Salvolini U, Scarabino T. High field MRI’, 2003. Eur J Radiol. 2003;48(2):137. 10.1016/j.ejrad.2003.08.011. 14680903 10.1016/j.ejrad.2003.08.011

[4] Westbrook C, Roth CK, Talbot J. In: MRI in practice fourth edition RT (R) (MR) (CT) (M) (CV), FSMRT CEO, imaging Educati on associates Pennsylvania USA’.

[5] Kwok WE. Basic principles of and practical guide to clinical MRI radiofrequency coils. Radiographics. 2022, May;42(3):898–918. 10.1148/rg.210110. 35394887 10.1148/rg.210110

[6] Kaza E, Klose U, Lotze M. Comparison of a 32-channel with a 12-channel head coil: Are there relevant improvements for functional imaging? J Magnetic Reson Imag. 2011, Jul;34(1):173–83. 10.1002/jmri.22614. 10.1002/jmri.22614 21618334

[7] Gruber B, Froeling M, Leiner T, Klomp DWJ. RF coils: a practical guide for nonphysicists. Magn Reson Imag. 2018;48(3):p. 590–604. 10.1002/jmri.26187. 10.1002/jmri.26187 PMC6175221 29897651

[8] May MW, Hansen S-LJD, Mahmutovic M, Scholz A, Kutscha N, Guerin B, et al. A patient-friendly 16-channel transmit/64-channel receive coil array for combined head–neck MRI at 7 Tesla. Magnetic Reson Med. 2022, Sep;88(3):1419–33. 10.1002/mrm.29288. 10.1002/mrm.29288 PMC9675905 35605167

[9] Kulkarni S, Kulkarni M, Patankar A, Watve A. Role of Double inversion recovery sequence in neuro-imaging on 3 Tesla MRI. Neurol India. 2021, Apr;69(2):394. 10.4103/0028-3886.314551. 33904461 10.4103/0028-3886.314551

[10] Wattjes MP, et al. ‘Double inversion recovery brain imaging at 3T: diagnostic value in the detection of multiple sclerosis lesions’. [Online]. Available: https://www.ajnr.org/PMC8134107 17213424

[11] Mardanshahi Z, Tayebi M, Shafiee S, Barzin M, Shafizad M, Alizadeh-Navaei R, et al. Evaluation of subacute subarachnoid haemorrhage detection using a magnetic resonance imaging sequence: double inversion recovery. Biomed (Taiwan). 2020;10(4):29–35. 10.37796/2211-8039.1058. 10.37796/2211-8039.1058 PMC7735974 33854932

[12] Soares BP, Porter SG, Saindane AM, Dehkharghani S, Desai NK. Utility of double inversion recovery MRI in paediatric epilepsy. 2016, British Institute of Radiology. doi: 10.1259/bjr.20150325. 10.1259/bjr.20150325 PMC4985945 26529229

[13] Bouman PM, Strijbis VI, Jonkman LE, Hulst HE, Geurts JJ, Steenwijk MD. Artificial double inversion recovery images for (juxta)cortical lesion visualization in multiple sclerosis. Mult Scler J. 2022, Apr;28(4):541–49. 10.1177/13524585211029860. 10.1177/13524585211029860 PMC8961242 34259591

[14] Costagli M, Lapucci C, Zacà D, Bruschi N, Schiavi S, Castellan L, et al. Improved detection of multiple sclerosis lesions with T2-prepared double inversion recovery at 3T. J Neuroimaging. 2022, Sep;32(5):902–09. 10.1111/jon.13021. 35776654 10.1111/jon.13021 PMC9544719

[15] Guan X, Zhang X, Yang H-J, Dharmakumar R. On the loss of image contrast in Double-inversion-recovery prepared T2 MRI of intramyocardial hemorrhage’, 2023. Magn Reson Imag. 2024;105:125–32. 10.1016/j.mri.2023.11.010. 10.1016/j.mri.2023.11.010 PMC13041346 37993042

[16] Parikh PT, Sandhu GS, Blackham KA, Coffey MD, Hsu D, Liu K, et al. Evaluation of image quality of a 32-channel versus a 12-channel head coil at 1.5T for MR imaging of the brain. AJNR Am J Neuroradiol. 2011, Feb;32(2):365–73. 10.3174/ajnr.A2297. 21163877 10.3174/ajnr.A2297 PMC7965710

[17] Hossein J, Fariborz F, Mehrnaz R, Babak R. Evaluation of diagnostic value and T2-weighted three-dimensional isotropic turbo spin-echo (3D-SPACE) image quality in comparison with T2-weighted two-dimensional turbo spin-echo (2D-TSE) sequences in lumbar spine MR imaging. Eur J Radiol Open. 2019, Jan;6:36–41. 10.1016/j.ejro.2018.12.003. 30619918 10.1016/j.ejro.2018.12.003 PMC6312863

[18] Montin E, Lattanzi R. Seeking a widely adoptable practical standard to estimate signal-to-noise ratio in magnetic resonance imaging for multiple-coil reconstructions. Magn Reson Imag. 2021, Jul;54(6):1952–64. 10.1002/jmri.27816. 10.1002/jmri.27816 PMC8633048 34219312

[19] Eichinger P, Hock A, Schön S, Preibisch C, Kirschke JS, Mühlau M, et al. Acceleration of Double inversion recovery sequences in multiple sclerosis with Compressed sensing. Invest Radiol. 2019, Jun;54(6):319–24. 10.1097/RLI.0000000000000550. 30720557 10.1097/RLI.0000000000000550

[20] Shrout PE, Fleiss JL. Intraclass correlations: uses in assessing rater reliability. Psychological Bull. 1979;86(2):420–28. 10.1037/0033-2909.86.2.420. 10.1037//0033-2909.86.2.420 18839484

[21] Kreitner K-F, Romaneehsen B, Krummenauer F, Oberholzer K, Müller LP, Düber C. Fast magnetic resonance imaging of the knee using a parallel acquisition technique (mSENSE): a prospective performance evaluation. Eur Radiol. 2006, Aug;16(8):1659–66. 10.1007/s00330-006-0288-0. 16733683 10.1007/s00330-006-0288-0

[22] Wright SM, Magin RL, Kelton JR. Arrays of mutually coupled receiver coils: theory and application. Magnetic Reson Med. 1991, Jan;17(1):252–68. 10.1002/mrm.1910170128. 10.1002/mrm.1910170128 2067400

[23] Keil B, Blau JN, Biber S, Hoecht P, Tountcheva V, Setsompop K, et al. A 64-channel 3T array coil for accelerated brain MRI. Magnetic Reson Med. 2013;70(1):248–58. 10.1002/mrm.24427. 10.1002/mrm.24427 PMC3538896 22851312

[24] Wiggins GC, Triantafyllou C, Potthast A, Reykowski A, Nittka M, Wald LL. 32-channel 3 Tesla receive-only phased-array head coil with soccer-ball element geometry. Magnetic Reson Med. 2006, Jul;56(1):216–23. 10.1002/mrm.20925. 10.1002/mrm.20925 16767762

[25] Manoliu A, Spinner G, Wyss M, Filli L, Erni S, Ettlin DA, et al. Comparison of a 32-channel head coil and a 2-channel surface coil for MR imaging of the temporomandibular joint at 3.0T. Dentomaxillofac RAD. 2016;45(4):20150420. 10.1259/dmfr.20150420. 10.1259/dmfr.20150420 PMC4846178 26837671

[26] Schmitt T, Rieger JW. Recommendations of choice of head coil and prescan normalize filter depend on region of Interest and task. Front. Neurosci. 2021, Oct;15. 10.3389/fnins.2021.735290. 10.3389/fnins.2021.735290 PMC8585748 34776844

[27] Walsh DO, Gmitro AF, Marcellin MW. Adaptive reconstruction of phased array MR imagery. Magn Reson Med. 2000, May;43(5):682–90. doi: 10.1002/(SICI)1522-2594(200005)43:5<682::AID-MRM10>3.0.CO;2-G. 10.1002/(sici)1522-2594(200005)43:5<682::aid-mrm10>3.0.co;2-g 10800033

[28] Bauer JS, Banerjee S, Henning TD, Krug R, Majumdar S, Link TM. Fast high-spatial-resolution MRI of the ankle with parallel imaging using GRAPPA at 3 T. Am J Roentgenol. 2007, Jul;189(1):240–45. 10.2214/AJR.07.2066. 17579177 10.2214/AJR.07.2066

[29] Fang Q, Yang Q, Wang B, Wen B, Xu G, He J. Enhancing lesion detection in inflammatory myelopathies: a deep learning–reconstructed double inversion recovery MRI approach. AJNR Am J Neuroradiol. 2025, Jun;46(6):1180–87. 10.3174/ajnr.A8582. 39542724 10.3174/ajnr.A8582 PMC12152806

[30] Bouhrara M, et al. Open access edited by variability and reproducibility of multi-echo T relaxometry: insights from multi-site, multi-session and multi-subject MRI acquisitions’. 10.3389/fradi.2022.930666 PMC10365099 37492668

[31] Xue C, Yuan J, Zhou Y, Wong OL, Cheung KY, Yu SK. Acquisition repeatability of MRI radiomics features in the head and neck: a dual-3D-sequence multi-scan study. Vis Comput Ind Biomed Art. 2022, Dec;5(1). 10.1186/s42492-022-00106-3. 10.1186/s42492-022-00106-3 PMC8971276 35359245

